# Additives in Nanocrystalline Tin Dioxide: Recent Progress in the Characterization of Materials for Gas Sensor Applications

**DOI:** 10.3390/ma16206733

**Published:** 2023-10-17

**Authors:** Darya Filatova, Marina Rumyantseva

**Affiliations:** Chemistry Department, Moscow State University, Moscow 119991, Russia; gak1.analyt@gmail.com

**Keywords:** tin dioxide, semiconductor gas sensor, nanocomposite, composition characterization

## Abstract

Tin dioxide has huge potential and is widely studied and used in different fields, including as a sensitive material in semiconductor gas sensors. The specificity of the chemical activity of tin dioxide in its interaction with the gas phase is achieved via the immobilization of various modifiers on the SnO_2_ surface. The type of additive, its concentration, and the distribution between the surface and the volume of SnO_2_ crystallites have a significant effect on semiconductor gas sensor characteristics, namely sensitivity and selectivity. This review discusses the recent approaches to analyzing the composition of SnO_2_-based nanocomposites (the gross quantitative elemental composition, phase composition, surface composition, electronic state of additives, and mutual distribution of the components) and systematizes experimental data obtained using a set of analytical methods for studying the concentration of additives on the surface and in the volume of SnO_2_ nanocrystals. The benefits and drawbacks of new approaches to the high-accuracy analysis of SnO_2_-based nanocomposites by ICP MS and TXRF methods are discussed.

## 1. Introduction

Tin dioxide SnO_2_ is an important representative of the group of *n*-type wide-bandgap (E_g_ = 3.6 eV) semiconductor oxides (SnO_2_, ZnO, In_2_O_3_, TiO_2_, and WO_3_ [1]) with a unique combination of high transparency, electrical conductivity, high surface reactivity, and stability in air. Tin dioxide has huge potential and is widely studied and used for applications in different fields [2,3], in particular for the manufacturing of optically passive components in a number of devices [4,5,6], including photodetectors [7,8,9,10,11,12], solar cells [13,14,15,16], conductive ceramics [17,18,19,20,21,22], varistors [23], transparent electrodes [24,25,26,27,28], electrochromic coatings [29,30,31,32,33,34], transistors [35,36,37], components of fuel cells [38] and metal-ion batteries [39], catalysts [40], and chemical sensors [2,41,42,43,44]. A wide range of applications require the development of technologies for producing tin dioxide with significantly different properties: high resistance for heaters, high conductivity for transparent electrodes and contacts, and a large specific surface area with selective adsorption properties and reactivity combined with appropriate conductivity for chemical sensors. It should be noted that bare tin dioxide without special additives does not have the abovementioned properties and is rarely used. For example, the required level of SnO_2_ electrical conductivity is achieved by introducing electroactive additives into the bulk of tin dioxide (doping), among which Sb, Nb, and F are the most widespread. The pentavalent Sb and Nb, which are close to Sn^4+^ in ionic radius, exhibit donor properties and increase the concentration of free charge carriers when replacing Sn^4+^ cations in the SnO_2_ crystal lattice, as well as fluorine F^−^ anions when replacing oxygen O^2−^ anions.

Considering the material requirements for semiconductor gas sensors, it should be noted that the stability of SnO_2_ in air is necessary to ensure long-term functioning at the elevated operating temperatures that are usually realized when detecting gases; a large specific surface area is needed to increase the proportion of material in direct contact with the gas phase; the reactivity of the surface determines the possibility of the adsorption of the target gas molecules and the occurrence of the reactions responsible for the sensor response formation; and a suitable level of electrical conductivity should make it possible to register the changes when interacting with gases, both electron donors and acceptors. Discussing the use of SnO_2_-based materials for gas sensor applications, it is worth noting that the phase composition, the chemical composition of the surface, the spatial distribution of the components, and the real structure of the materials are determined by the synthesis conditions. In turn, these characteristics determine the reactivity of the materials and the mechanism of their interaction with the gas phase; the concentration and mobility of the charge carriers; and, consequently, the sensor parameters, namely sensitivity, selectivity, and stability. The specificity of the chemical activity of tin dioxide in the interaction with the gas phase is achieved via the immobilization of various modifiers on the SnO_2_ surface: clusters or nanoparticles of precious metals and their oxides (Au, PtO_x_, PdO_x_, and RuO_x_) or transition metal oxides (La_2_O_3_, Fe_2_O_3_, Co_3_O_4_, NiO, CuO, etc.) [45,46,47,48,49,50,51]. Modifiers affect the concentration of adsorption centers and the mechanism of reactions on the SnO_2_ surface [51]. The specificity of adsorption and the reduction in the reaction temperature for modified SnO_2_ are the result of a synergistic effect of a number of factors: the formation of acidic or basic centers; the activation of chemisorbed molecules; and, in some cases, the spillover effect [46,51,52]. The conscious choice of the modifier can be based on fundamental ideas about the acid–base properties of the catalytic modifier quantified via the value of the optical basicity [53,54,55] or the Sanderson electronegativity scale [56,57] used in heterogeneous catalysis for analyzing the adsorption and reaction activity of materials [46].

Complex inhomogeneous nanocrystalline systems—nanocomposites formed by introducing additives into a highly dispersed tin dioxide matrix [45,46,47,48,49,50,51,52]—are systems constructed of nanometer-sized crystalline particles (3–30 nm) combined into agglomerates, in which the additive is distributed in a complex way between the volume and the surface of the crystalline grains of the main phase [58]. Considering that in nanocrystalline systems (which are non-equilibrium systems), surface atoms make a significant contribution to the interaction of the main phase and the modifier, it is impossible to predict the nature of the mutual distribution of components in such systems using equilibrium P-T-x phase diagrams. In Gibbs’s approach, interface phases (including the solid/gas interface) are modeled by the combination of a phase possessing an area but no volume (a dividing surface) with surface excess properties accounting for all the effects of the interface [59]. By mathematically uniquely placing the dividing surface, all thermodynamic variables of the surface are accounted for in a way that captures all the effects of the interface. This is an important concept to understand because it is not to say that the approach of Gibbs can only be applied to interfaces that have no reach into the adjoining bulk phases, but rather that the excess molecules and energy of that reach must be added to the presence of a uniquely defined infinitely thin surface to completely describe the system. In bulk systems, the surface effect is negligible, so the contribution of the surface thermodynamic functions to the thermodynamic description of the system as a whole (graphically represented by P-T-x phase diagrams) is absent. This makes equilibrium P-T-x phase diagrams inapplicable for describing highly dispersed systems, including nanocrystalline solids. For example, it has been shown that, unlike microcrystalline systems, the regions of the existence of solid solutions and the conditions of phase formation in nanocrystalline materials significantly depend on the dispersion of samples [45]. It was established in [45,48,60] that the introduction of additives leads to an increase in the thermal stability of nanocomposites compared with individual oxides. Due to the significant proportion of surface layers with a high degree of structural disordering, grain growth in nanocrystalline systems is accompanied by the structural relaxation of the surface. The release of an additive in the form of the segregation of an amorphous phase on the surface of growing SnO_2_ crystallites slows down the rate of their growth under isothermal annealing [45] due to the so-called Zener pinning of the matrix grain boundaries [61], according to which the maximum size of a crystalline grain is determined by the volume fraction and size of particles segregated on the surface of the growing crystallites. Depending on the ratio of components, nanocomposites also have different phase compositions. In addition to the formation of solid solutions, the formation of additive segregation on the surface of the crystalline grains of the main phase and the stabilization of thermodynamically unstable phases are possible [62].

Experimental studies on “composition–structure–property” relationships indicate that the additives that are used to achieve the necessary functional parameters of tin dioxide, depending on the concentration and local position in the bulk or on the surface, perform the function of electroactive dopants or electrical/chemical active modifiers. The effect that additives have on SnO_2_’s properties primarily depends on the element’s electronic structure, structural parameters, and acid/base and redox properties. Given that additives are distributed in a complex way between the surface and the bulk of SnO_2_ crystallites, the implementation of only one function is impossible. In turn, the additive’s distribution between the surface and the volume of SnO_2_ crystallites has a significant effect on the sensor’s characteristics, namely sensitivity and selectivity.

Depending on the electronic structure of the atoms, the additives that have been most used for SnO_2_ functionalization can be divided into the following groups:

- *p*-elements—Sb and F;
- 3*d*-, 4*d*-, and 5*d*-elements—Ti-Zn, Nb, Mo, and W;
- Rare earth elements—La and Ce;
- Precious metals and platinum group metals—Pt, Pd, Ru, Rh, Au, and Ag.

The complexity of interpreting the dependence of the functional properties on the nature and concentration of the introduced additive is caused by the fact that most of them contain elements that can exhibit different electronic states in the volume and on the surface of SnO_2_ crystallites [63,64,65,66,67]. Thus, the correct determination of the composition of the nanocomposite is necessary for the development of technology to obtain functional materials based on tin dioxide. It should be noted that in the case of nanocomposites, the term “composition” is complex and includes the gross quantitative elemental composition; phase composition; surface composition; electronic state of the additives and main components; and distribution of the additives between the volume and surface of the SnO_2_ crystallites.

Currently, there is no single technique devoted to the creation of materials for gas sensors that allows for the conscious choice of an additive in order to selectively increase the sensitivity of semiconductor oxides to a certain type of detected gas. The most promising, in our opinion, are catalytic approaches based on the concepts of “collective” and “local” centers. The consideration within the framework of the “collective” centers approach is based on the theory of chemisorption on the surface of semiconductors, developed in the works of T. Wolkenstein [68], which gives an idea of the mechanism of the adsorbate effect on the overall band structure of the modified semiconductor matrix. In this case, the catalytic activity of the modifier is directly related to the electronic state of the doping additives in the oxide matrix and their effect on the concentration of charge carriers in the semiconductor. On the other hand, the approach of “local” centers is based on the concept of an inhomogeneous surface. The interaction of the semiconductor oxide with the gas phase in this case is described through the formation of surface complexes, while the chemical nature of the modifier and its reactivity in acid/base or redox reactions play a decisive role. It is obvious that the regularities established in the study of catalysts may not be observed when applied to sensor materials and should be tested experimentally. This requires, among other things, a detailed characterization of semiconductor materials in order to reliably establish the patterns of “composition–structure–functional property” relationships.

This review discusses recent approaches to analyzing the composition of SnO_2_-based nanocomposites that act as sensitive materials for semiconductor gas sensors and systematizes the experimental data obtained by various authors using a set of modern analytical methods for studying the concentration and distribution of additives between the surface and volume of SnO_2_ crystallites. A comparison of the characteristics of the analytical methods discussed in this review is presented in Table 1. Diagrams illustrating the analytical dimension against the detection range and depth of analysis can be found in [69].

## 2. Gross Quantitative Elemental Composition

Despite the huge amount of experimental data on the effect of the concentration of additives on SnO_2_ sensor properties, a strategy for the quantitative elemental analysis of tin-dioxide-based nanocomposites has not been proposed. To characterize nanocrystalline SnO_2_, approaches that are convenient for analyzing the surface of solid samples and thin films are more often used; therefore, X-ray methods have become widespread. The chemical composition and quantitative ratio of components for nanoscale functional materials are most often determined by energy-dispersive X-ray spectroscopy (EDX) techniques in combination with electron microscopy. This approach is actively developing [70].

Energy-dispersive X-ray spectroscopy (EDX) is an analysis method that can be coupled with several applications, including SEM, TEM, and STEM. EDX, when combined with an electron microscope, can provide elemental analysis for areas as small as several nanometers. The effect of the electron beam on the surface and its penetration into the particles produces X-rays that are characteristic of the elements found on and near the surface of the sample. Using the EDX method, one can determine the elemental composition of individual points or map out the areal distribution of elements based on the scanning capability of the electron microscope. EDX does not fit into the category of techniques for surface science, as the X-rays are generated in a region about 2 microns in depth [69].

The accuracy of quantitative analysis by the EDX method is influenced by the porosity of the sample, its micro-uniformity (the presence of two or more phases), the material density, the peak overlap, the matrix composition, and the amount of additive. For analysis by the EDX method, it is necessary to choose a sample thickness that is, on the one hand, sufficient to ensure a good signal-to-noise ratio and, at the same time, thin enough to reduce the effects of electron scattering [71].

The advantage of X-ray spectra, in comparison with the spectra in the visible and ultraviolet regions, is the small number of lines for each element. When an atom with a vacancy passes from the excited to the ground state, an Auger electron can be emitted instead of X-ray characteristic photons. The probability of the emission of characteristic X-rays increases with an increasing atomic number, while the probability of the emission of Auger electrons decreases. Thus, the EDX method is most useful for the analysis of heavy elements, especially when the element concentration is low. The area of interaction between electrons and matter increases rapidly with the energy of the incident electron beam. The penetration depth decreases with an increasing material density. Dense metals, such as W and Au, have a limited electron transmission region, and, consequently, EDX spectra are more sensitive only for the surface layer of a sample of such materials. The results of quantitative X-ray analysis can be achieved with an accuracy of up to 1%. These results require careful analysis and should be obtained using standard samples. Most EDX computer programs work using quantification without standards, though this does not always lead to a reliable result. The most common method of quantification is known as the three-correction method. This method consists in measuring the X-ray signal I from an element in an unknown sample, and then the data obtained are compared with the peak signal for the same element in the reference sample, taking into account the correction coefficients: the atomic number, absorption, and fluorescence correction. It is important to note that any method of calculation assumes that the sample is flat and homogeneous in the volume excited by X-ray radiation. If X-rays are emitted from an area with a different composition or from an uneven surface, the absorption and fluorescence corrections may be inaccurate [72].

The geometry of the samples can also introduce significant uncertainty into the results of quantitative analysis. An interesting way to account for this factor by collecting information using several detectors arranged symmetrically relative to the sample was proposed by the authors of [73]. Some of the factors affecting the accuracy of the analysis can be taken into account using standard samples with the same surface conditions; however, there are no such samples for new materials. These difficulties can lead to an incorrect interpretation of the obtained dependencies of various material characteristics on the composition determined by the EDX method.

For example, for single-phase (according to XRD data) SnO_2_ samples doped with Fe by co-precipitation, the dependences of the cell parameters, volume of the unit cell, and sizes of the SnO_2_ crystallites on the concentration of added iron were shown in [74]. In this case, the sensitivity of the EDX method was sufficient to detect iron in the concentration range used, and the step of changing the concentration by 3–5 wt.% allowed the authors to correlate the structural characteristics with the iron content, but without evaluating the accuracy and locality of these results. It should also be noted that the results, obtained in this work by ^57^Fe Mössbauer spectroscopy, indicated the release of some Fe^3+^ cations into amorphous α-Fe_2_O_3_ on the SnO_2_ crystallites’ surface, but no estimates of the concentration of Fe^3+^ on the surface and in the volume of the SnO_2_ grains were given. The authors of [75], when characterizing SnO_2_ microtubules synthesized by the catalyst-free vapor–solid (VS) thermal method, discussed the differences in the results of the EDX analysis of the chromium content on the inner and outer surfaces of the tubes at the level of 1.0 and 1.2 at%, respectively, while there is no reason to believe that these results belong to different confidence intervals.

The reliability of determining the additive concentration at the level of 1–5 wt.% by the EDX method is often questionable. For example, in [76], to study the purity of the chemical composition of Co-doped SnO_2_ nanoparticles obtained via the hydrothermal method, samples were analyzed using EDX. In this case, the cobalt content in the material at a level of less than 1 wt.%, determined by the internal normalization method, was only an approximate estimate. In [77], 0.5, 1, 2, and 3 wt.% Pd-loaded 6 mol% Sm-doped SnO_2_ nanoparticles prepared using a co-precipitation method were investigated. For a composite with a pre-assigned Pd content of 3 wt.%, a 2.13 wt.% Pd content was found by the EDX method, but the accuracy of the results to hundredths of a percent was not confirmed. With an increase in the concentration of the additive, the EDX signal could be reliably detected, and the results obtained could be used to assess the composition of the samples. Given that the accuracy of the EDX determination of light elements, in particular oxygen, is low, relative ratios are often used to determine the concentration of additives in oxide systems, which can be estimated by the fundamental parameter method [78,79]. There are very few data verifying the correctness of the results of composition analysis by the EDX method. Thus, the authors of [80], for SnO_2_-Ag nanocomposites obtained by the sol–gel method, confirmed the results of EDX determination (3.7 wt.% Ag) by ICP-MS (4.1 wt.% Ag), while the method of sample dissolution was not specified. In [81], for a correct analysis of the elemental composition of SnO_2_/Fe_2_O_3_ nanocomposites by the EDX method, ceramic samples with different [Fe]/[Sn] ratios prepared from appropriate mechanical mixtures of oxides were used as reference materials.

## 3. Phase Composition

The analysis of the phase composition of nanocrystalline materials is a difficult task because of the significant broadening of the diffraction maxima; furthermore, traditional X-ray diffraction studies require the use of additional methods.

In SnO_2_-based nanocomposites, the crystalline phase containing the additive is often detected only at a sufficiently high concentration of the introduced additive and high annealing temperatures; in other cases, it is possible to form a solid solution and/or segregation of the amorphous phase containing the additive on the surface of the SnO_2_ crystallites. The implementation of one or the other option is determined by both fundamental reasons—for example, the difference in the ionic radii of Sn^4+^ (0.69 Å) and the additive’s cation M^n+^—and the synthesis conditions—the method of introducing the additive, the concentration of the additive, and the temperature treatment. Based on the values of the ionic radii of metal cations in an oxygen octahedral environment [82], it can be assumed that the formation of SnO_2_-based solid solutions is possible upon the introduction of Sb^5+^ (0.60 Å) [83,84,85,86]; Ti^4+^ (0.605 Å) [87,88,89]; Cr^3+^ (0.615 Å) [65,90]; Mn^3+^ (0.645 Å, high spin) [64,91,92,93]; Fe^3+^ (0.645 Å, high spin) [81,94,95,96,97]; Co^2+^ (0.65 Å, low spin) [63,95,96,98,99,100,101]; Ni^2+^ (0.69 Å) [95,96,102,103,104,105,106]; Cu^2+^ (0.73 Å) [107,108,109]; Zn^2+^ (0.745 Å) [110,111,112]; Nb^5+^ (0.64 Å) [113,114,115]; and Ru^3+^ (0.68 Å) [67,116,117,118,119,120]. However, it is unlikely in the case of Pd^2+^ (0.86 Å) [121,122,123,124,125,126]; Pt^2+^ (0.80 Å) [127,128]; La^3+^ (1.06 Å) [129,130]; and Ce^4+^ (0.80 Å) [131,132,133]. An unambiguous conclusion about the formation of a solid solution can be made if there is a change in the parameters of the SnO_2_ crystal lattice with an increase in the concentration of the additive. However, in nanocrystalline systems, the exact determination of cell parameters from X-ray data is often difficult because of the significant broadening of the diffraction maxima. For this reason, valuable information about the phase composition of SnO_2_-based nanocomposites can additionally be obtained by transmission electron microscopy, electron diffraction, Raman spectroscopy, and Mössbauer spectroscopy [45,62,63,64,81,94,134,135].

**Figure 1:**
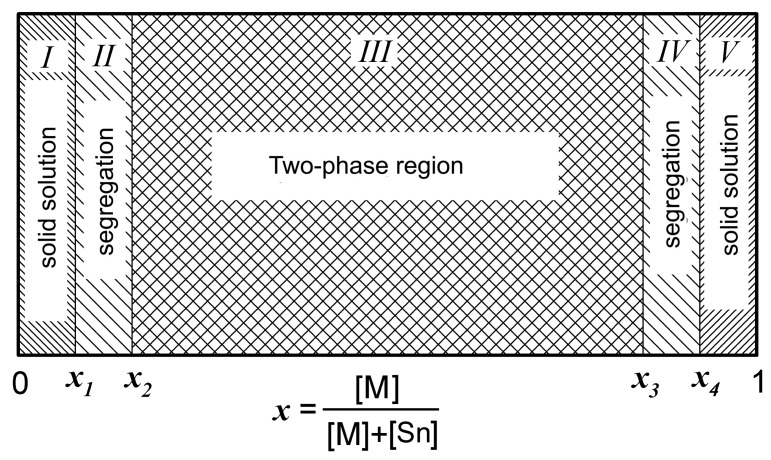
Scheme of the transformation of the phase composition for SnO_2_–M_n_O_m_ nanocomposites [45].

Based on a large set of experimental data, a scheme describing the transformation of the phase composition of tin-dioxide-based nanocomposites with an increase in the concentration of the introduced additive was proposed in the review [45] (Figure 1). This idea was later extended to metal oxide nanocomposites formed by two metals, M^I^ and M^II^, in the review [48]. With an increase in the additive content in SnO_2_-based nanocomposites, the formation of a solid solution based on tin dioxide, the segregation of the oxide of the second component in the form of a monolayer or islands on the surface of the crystallites of the main phase, and then the transition to a two-phase region were consistently observed. The positions of the boundaries *x*_1_, *x*_2_, *x*_3_, and *x*_4_ (Figure 1) are determined by the ratio of the ionic radii of M^n+^ and Sn^4+^ and the defectiveness of the SnO_2_ structure. The extent of the areas of solid solutions is units of percents. Determining the position of the *x*_1_ and *x*_4_ boundaries is an extremely difficult task and requires the development of special analytical procedures. With an increase in the annealing temperature, the defectiveness of the SnO_2_ structure decreases, which leads to a decrease in the solubility of the second component.

The synthesis method influences the distribution of the additive between the volume and surface of the crystalline grains of SnO_2_ due to the nonequilibrium of nanocrystalline systems. Thus, the introduction of an additive into a preformed SnO_2_ matrix (impregnation, layer deposition, etc.) leads to its accumulation on the surface of the SnO_2_ crystallites (Table 2). This leads to the supersaturation of the additive’s precursor on the surface of the SnO_2_ matrix and provides the possibility of forming the phase of the second component through heterogeneous nucleation. Since diffusion in the solid phase is slow, kinetic difficulties do not allow the second component to fully penetrate into the crystal structure of the main phase. This is an additional parameter that facilitates the crystallization of the second component in the form of its own oxide phase. The use of methods involving the synthesis of multicomponent nanocrystalline materials from a common source containing tin and additive precursors (co-deposition from the liquid phase, hydrothermal synthesis, the pyrolysis of aerosols, flame spray pyrolysis, etc.) (Table 3) provides a high degree of component homogenization, short diffusion paths, and the absence of conditions for the supersaturation and nucleation of the phase of the second component. The combination of these factors benefits solid solution formation.

The effect of the synthesis method on the phase composition of SnO_2_/NiO materials can be illustrated by comparing the results obtained in [102,158] for SnO_2_−NiO core−shell nanowires produced in a two-step process (single crystalline SnO_2_-core nanowires were synthesized by vapor−liquid−solid deposition and then decorated with a polycrystalline NiO-shell layer by atomic layer deposition) [158] and those obtained for Ni-doped SnO_2_ nanorods produced by one-pot solvothermal synthesis in [102]. In the first case, a conformal coating of a polycrystalline NiO-shell layer (2–8.2 nm thickness) onto single crystalline SnO_2_ nanowires was confirmed by grazing incidence X-ray diffraction (GIXRD) and high-angle annular dark-field scanning transmission electron microscopy (HAADF-STEM) with EDX mapping. The authors of [158] did not discuss the possibility of the formation of solid solutions near the SnO_2_/NiO boundary. On the contrary, one-pot solvothermal synthesis allowed the authors of [102] to obtain Ni-doped porous SnO_2_ materials. For samples with the composition set during the synthesis as [Ni]/[Sn] = 0, 5, 10, and 15 mol.%, the unit cell parameters were analyzed by Rietveld refinement. It was found that when the [Ni]/[Sn] ratio was set at less than 10 mol.%, the unit cell parameters decreased with the increase in the Ni^2+^ concentration. However, for the sample with [Ni]/[Sn] = 15 mol.%, the unit cell parameter increased. The authors attributed such a change in the unit cell parameters to the achievement of the Ni^2+^ solubility limit in the form of substitution defects in the SnO_2_ structure. Unfortunately, there was no information in the article about the Ni^2+^ concentration in the SnO_2_ bulk; however, it was indicated that the actual Ni content in the samples determined by the XPS method varied from 1.8 to 6.3 mol.%. Given that XPS has a small depth of analysis (Table 1), the values obtained may have been overestimated due to nickel segregation on the surface of the SnO_2_ crystalline grains.

Quantitative XRD phase analysis [170] is based on comparing the relative intensities of diffraction maxima using the Reference Intensity Ratio (RIR)—the ratio of the absolute intensities of the most intense reflections of a given substance and corundum (α-Al_2_O_3_) on a diffractogram of a mixture containing 50 wt.% of components. The sensitivity of the analysis varies for different phases, and the minimum detectable concentration is 5–15 wt.%. This approach is rarely used [81,171] and has no practical application for the analysis of nanocrystalline materials since it requires additional calibration using other analysis methods.

## 4. Surface Composition and Electronic State of Additives

Information about the valence states of the elements, the chemical composition of the surface, and the interfaces that determine the properties of nanocrystalline materials can be obtained by X-ray photoelectron spectroscopy (XPS) [172,173]. The XPS method allows one to detect all elements except hydrogen. The detection limit is usually 0.1–1 at.%, and the accuracy is 0.3–1 at.%. XPS is usually not considered a method that provides a lateral resolution in the nanometer range; however, the depth of analysis is determined by the electron free path relative to inelastic collisions and is 0.5–2.5 nm for metals and 4–10 nm for organic substances. Conventional XPS operates at vacuum levels of about 10^−9^ mbar, since the signal intensity decreases because of the higher electron scattering losses within the gas or liquid medium.

The study of the fine structure of the O1s oxygen spectrum on the surface of nanocrystalline materials based on SnO_2_ allows one to evaluate the effect of the introduced modifiers on the number of chemisorbed oxygen-containing particles that can act as the Lewis or Brønsted base when interacting with gas-phase molecules [51]. The asymmetric O1s signal of nanocrystalline SnO_2_-based materials can usually be simulated by two components: the major signal with a binding energy of 530.0–530.5 eV due to bulk O^2−^ anions and the lower peak of surface oxygen-containing species (various forms of chemisorbed oxygen and surface hydroxogroups) at higher a binding energy (531.0–533.0 eV). Determining the ratio between the intensities of the spectrum components corresponding to bulk O^2−^ anions and surface oxygen species allows one to draw conclusions about the effect of additives on the concentration of the basic and oxidizing centers on the surface of SnO_2_-based materials and establish appropriate relationships with their sensor properties when detecting gases of various natures [42,45,46,51,63,64].

The influence of the predominant form of chemisorbed oxygen on the sensitivity of semiconductor sensors based on nanocrystalline SnO_2_ was theoretically justified in [174]. This effect is expressed in a change in sensitivity—the slope of the dependence of the sensor response on the partial pressure of the detected reducing gas, drawn in double logarithmic coordinates. Experimentally, the effect of the additive on the predominant form of chemisorbed oxygen and sensitivity when detecting CO and H_2_ was established for SnO_2_/SiO_2_ materials [175,176,177]. For SnO_2_/SiO_2_ core–shell nanofibers obtained via template synthesis, under the introduction of water vapor at 400 °C, the predominant oxygen species remained both O^2−^ and O^−^. Meanwhile, under the same conditions, the dominant oxygen species on the SnO_2_ nanoparticles were changed from O^2−^ to O^−^, which was accompanied by a decrease in sensitivity when detecting H_2_ [175]. For SnO_2_/SiO_2_ nanocomposites synthesized by the hydrothermal method, an increase in the silicon content led to an increase in the contribution of molecular O_2_^−^ ions that was consistent with the data obtained by EPR spectroscopy [176]. This change in the predominant form of chemisorbed oxygen was accompanied by an increase in sensitivity and a decrease in the CO detection limit, including in humid air [177].

The results of the quantitative analysis of SnO_2_-based nanocrystalline material composition by the XPS method are rarely discussed. Thus, the ratio of photoelectronic line intensities was used to approximate the ratio of Ru and Sb on the surface of SnO_2_ films in [178]. The authors of [179] obtained results for the determination of the Sb content on the surface of SnO_2_ synthesized by the vapor–liquid–solid process, while the inclusion of antimony in the SnO_2_ lattice was confirmed by Raman spectroscopy. The authors presented the antimony concentration with an accuracy of hundredths of a percent (8.19 at.%) but did not indicate the error of determination, and the relationship between the obtained quantitative results and the properties of the semiconductor nanowires was not discussed.

When analyzing the active centers created in gas-sensitive SnO_2_-based nanocrystalline materials, it is important to determine the oxidative state of the introduced additive. For the noble metals used, the tendency to form oxides decreases in the raw: Ru, Rh, Pd, Pt, Au. A combination of XPS and EPR methods showed that ruthenium in SnO_2_/RuO_y_ nanocomposites (1 wt.% Ru), obtained by SnO_2_ impregnation with Ru(acac)_3_ solution and subsequent thermal annealing, could be in a mixed-valence state due to the formation of RuO_2_ clusters, including a fraction of Ru^3+^ [67,116]. Gold, even in the form of small clusters (1–3 nm) on the surface of sensor materials, is usually present in the zero–valent state [180,181,182]. However, components corresponding to Au^3+^ were found in the Au4f XP spectra of SnO_2_/SiO_2_–Au nanocomposites, which was explained by the ability of Au^3+^ (0.68 Å) to be embedded in the positions of coordination-unsaturated tin cations present on the partially reduced surface of SnO_2_ [183]. An additional effect exerted by SiO_2_ nanoparticles is the stabilization of Au cationic forms by hydroxyl groups due to the formation of Au^δ+^−OH bonds [184], with silanol groups making a significant contribution to the formation of these bonds.

Palladium and platinum can both be in the metallic state and in the form of PdO, PtO, and PtO_2_ oxides. The oxidation degree in this case depends on the nanocomposites’ synthesis procedure, the morphology and size of the additive particles, and the temperature and composition of the surrounding gas phase. XPS studies of the state of palladium deposited on SnO_2_ have shown that in low concentrations (0.1–0.2 wt.%), the modifier is distributed over the surface at the atomic level, which facilitates its interaction with chemisorbed oxygen to form PdO [121,122]. With an increase in the content to 1–3 wt.%, palladium forms three-dimensional clusters on the SnO_2_ surface [121], which in the presence of oxygen already begin to oxidize at 200 °C. As a result, mixed-valence PdO_x_ clusters are formed containing Pd^2+^ as the main form (x = 0.7—0.8) along with zero-valent palladium [122,123,124]. Pd^2+^ cations can be embedded in the SnO_2_ lattice at the Sn^4+^ positions [121]; however, heating above 400 °C is required to start Pd^2+^ diffusion into the near-surface layer of the SnO_2_ matrix [125]. A detailed study of the composition of SnO_2_/PdO_x_ (1 wt.% Pd) nanocomposites by a complex of methods (XPS, EPR, XANES, and EXAFS) revealed that PdO_x_ clusters consisting of amorphous PdO also included Pd^0^ atoms and a small fraction of Pd^3+^ cations, presumably stabilized at the boundary of the clusters and the SnO_2_ matrix [67,116]. A further increase in the amount of palladium deposited led to the percolation of metal clusters; the formation of continuous Pd layers; and, finally, a near-surface solid solution of Sn–Pd (2–5 monolayer additives) [121]. The XPS studies carried out by the authors of [126] showed that in SnO_x_-decorated Pd nanocubes (the estimated edge length of the cubes was 21 ± 7 nm), palladium was mainly present as Pd^0^, but a small amount of PdO and PdO_2_ was also detected. The XPS results did not indicate the intermixing of Pd and SnO_2_ and the formation of an interfacial PdSn alloy.

In a similar way, the particle size affects the state of platinum on the surface of nanocrystalline SnO_2_: it has been shown that clusters smaller than 3–4 nm represent PtO_2_, and larger ones represent Pt^0^ [127]. According to XPS, in SnO_2_/PtO_x_ (1 wt.% Pt) nanocomposites obtained by SnO_2_ impregnation with Pt(acac)_2_ and subsequent thermal annealing, platinum presents as PtO, which is typical for small (less than 2 nm) Pt clusters. In the case of a SnO_2_/PtPdO_x_ nanocomposite containing bimetallic PdPtO_x_ clusters, the Pd3d XP spectrum was shifted toward lower energies, indicating the partial reduction of Pd, while the Pt4f XP spectrum corresponded to the Pt^2+^ oxidation state in PtO [128].

The unambiguous determination of the electronic state of 3D elements by the XPS method can be difficult due to the similar electron binding energies for different electronic states [185]. For example, in the Ti2p XP spectra of hollow TiO_2_-SnO_2_-TiO_2_ composite nanofibers (the body consisted of hollow SnO_2_ nanofibers, fabricated by a facile electrospinning technique, and the inner and outer surfaces were covered by mixed-phase TiO_2_ nanoparticles grown via the hydrothermal method) [186], the Ti 2p_3/2_ peak was broad and asymmetric, demonstrating that there were at least two kinds of Ti chemical states. After curve fitting, besides the peak corresponding to Ti^4+^, another peak with a lower binding energy was identified, suggesting the existence of Ti^3+^ (or Ti–SnO bonds at the interface between the TiO_2_ nanoparticles and SnO_2_ nanoparticles) in the composite.

In SnO_2_/MnO_x_ nanocomposites ([Mn]/[Sn] = 1 at.%) obtained by SnO_2_ impregnation with Mn(acac)_3_ and subsequent thermal annealing [64], the manganese oxidation state could not be unambiguously determined from the Mn2p XP spectra, since the difference between the electron bond energy of Mn 2p_3/2_ peaks for Mn^3+^ and Mn^4+^ is only 0.5–1 eV [187,188]. Additional information could be obtained by Raman spectroscopy.

The Co2p XP spectra of SnO_2_/CoO_x_ nanocomposites obtained by SnO_2_ impregnation with Co(NO_3_)_2_ and subsequent thermal annealing, depending on the total cobalt content in the nanocomposites and the annealing temperature of the SnO_2_ matrix, could be described by two or four doublets corresponding to Co^3+^ and Co^2+^ and satellite peaks of Co^2+^. In nanocomposites with a low annealing temperature (300 °C) for the SnO_2_ matrix, cobalt presented in the Co^2+^ oxidation state. In nanocomposites with a high annealing temperature (750 °C) for the SnO_2_ matrix with a total cobalt content of 1.5 at.%, only Co^2+^ was found, and an increase in the cobalt content led to the appearance of the Co^3+^ state [63]. Additional information obtained from EPR data made it possible to determine the fraction of paramagnetic Co^2+^ centers from the total cobalt content. Similarly, in Co-doped SnO_2_ nanoparticles (with a [Co]/[Sn] ratio of about 2 at.%) obtained via the hydrothermal method [76], cobalt presented only in the Co^2+^ state. In [101], cobalt-doped 3D inverse-opal SnO_2_ multilayer films were prepared using the ultrasonic nebulizing deposition (UND) method combined with a self-assembly template. The Co2p XP spectra were described using the Co^3+^ and Co^2+^ components, and the [Co^3+^]/[Co^2+^] ratio decreased with an increased total cobalt concentration. For cobalt-doped SnO_2_ nanobelts prepared by the chemical vapor deposition method [189] (the total cobalt content was not specified), the Co2p XP spectra did not allow us to make an unambiguous conclusion about the electronic state of the cobalt; however, based on a combination of results obtained by XRD and Raman spectroscopy, the authors of [189] claimed that Co^2+^ and Co^3+^ were successfully substituted into the SnO_2_ structure at the Sn^4+^ site. Note that this statement is controversial because of the significant difference in the ionic radii of Co^2+^ and Co^3+^.

Establishing the electronic state of 4f elements is an even more difficult task due to the complexity of XP spectra. However, the authors of [131] were able to identify certain patterns in the electronic state of cerium in CeO_2_-SnO_2_ nanocomposites. The results showed that the Ce^3+^ content in CeO_2_ was substantially lower than that of the CeO_2_-SnO_2_ composites, but the Ce^3+^ content was almost independent of the total Ce content. This indicates that the oxidation states of Ce are markedly affected by the presence of surrounding Sn species.

## 5. Distribution of Additives between the Volume and the Surface of SnO_2_ Crystallites

The distribution of the additive between the volume and the surface of the crystalline grains of the main phase plays an extremely important role in the formation of the functional properties of nanocomposites. The introduction of impurity atoms into the SnO_2_ crystal structure causes the formation of impurity levels, the compensation of donor oxygen vacancies by acceptor impurity defects, and the modulation of the band relief of the semiconductor. The segregation of the phase formed by the additive on the surface of SnO_2_ crystallites can lead to the formation of *p*–*n* heterojunctions in the area of intergranular contacts, which also inevitably affects the electrophysical properties of the material. In addition, the distribution of impurities between the volume and the surface of the SnO_2_ crystalline grains determines its effectiveness in the interaction between the material and the gas phase. The combination of these factors has a significant impact on the sensor properties of nanocomposites. Thus, the search for approaches to the correct highly sensitive determination of the additive concentration in the volume and on the surface of the SnO_2_ crystalline grains is an urgent task.

The formation of clusters or nanoparticles of noble metals or their oxides localized on the surface of SnO_2_ has been experimentally confirmed by the study of nanocomposites via transmission electron microscopy [190,191,192,193,194]. It is possible to detect clusters if the atomic masses of the additive and the main element of the oxide matrix differ significantly (for example, when tin dioxide is modified with gold or nickel), or if the sizes of the modifier clusters differ significantly from the SnO_2_ particle size. On the contrary, when noble metals of the same period (Ru, Rh, Pd) are immobilized on the surface of nanocrystalline SnO_2_ (particle size less than 10 nm), the visualization of additives by transmission microscopy methods is difficult [124,158]. When studying the distribution of additives in such systems, it is informative to scan the elemental composition of the surface using energy-dispersive X-ray spectral microanalysis (EDX mapping). For SnO_2_/PdO_x_ and SnO_2_/RuO_x_ nanocomposites, it has thus been shown that these additives form clusters of 1–5 nm even on highly dispersed SnO_2_ (particle size 3–6 nm) [124,190,193]. In general, the combination of transmission microscopy methods with EDX mapping makes it possible to visualize the distribution of components in nanocrystalline materials, as well as to analyze the phase composition with high locality [124,131,137,158,171,186,195]. However, the insufficient sensitivity of the EDX method often does not allow one to reliably determine the concentration of the additive embedded in the SnO_2_ crystal structure.

The solubility of antimony in nanocrystalline tin dioxide is estimated as 3–6 at.% based on the dependence of the conductivity on the Sb concentration [195,196,197,198]. Diffraction analysis methods are ineffective for studying antimony distribution in nanocrystalline SnO_2_. Due to the similar size of Sb^5+^ and Sn^4+^ (0.65 Å and 0.69 Å, respectively), cation substitution does not affect the unit cell parameters. Because of the proximity of the atomic masses of Sn and Sb, it is not possible to visualize the impurity in SnO_2_(Sb) by contrast using electron microscopy methods. According to Mössbauer spectroscopy, EXAFS, and XANES data, at low concentrations, antimony is embedded in the SnO_2_ structure in the Sb^5+^ form, and when the doping level increases, it comes to the surface in the Sb^3+^ form [66].

Fabbri et al. used a comparison of the results obtained by the EDX and XPS methods to estimate the distribution of the Sb additive between the volume and the surface of SnO_2_ nanocrystals based on different signal acquisition depths [199]. However, it remains unclear what factors were taken into account when calculating the Sb content with an accuracy of tenths of a percent. Zaytsev et al. used Auger electron spectroscopy to study the distribution of antimony between the surface and volume of SnO_2_ whiskers synthesized by the vapor–liquid–crystal method [200]. Auger spectra were obtained on the surface and after the ion beam etching of a 100 nm layer on the same whisker. Antimony was clearly visible on the surface, and after etching, no antimony signal was detected in the spectrum; thus, it was shown that antimony was concentrated in the near-surface layer of the whiskers (the layer thickness did not exceed 100 nm). At the same time, the antimony content in the volume of the whiskers was below the detection limit of the Auger spectroscopy method, and the antimony content found on the surface significantly exceeded its total amount determined by laser-induced mass spectrometry.

The authors of [201] proposed for the first time the use of the highly sensitive ICP MS method for the determination of antimony in SnO_2_ whiskers, representing a new approach to studying the distribution of the additive between the surface and volume. The approach included the treatment of the whisker surface with citric acid to transfer Sb^3+^ from the surface to stable soluble complexes, the complete dissolution of the sample residue after flushing from the surface, and the dissolution of the initial sample followed by the analysis of all solutions using the ICP MS method. The uniqueness of the proposed approach was based on SnO_2_’s properties: the material is barely soluble, so it was possible to select a reagent for flushing the additive from the SnO_2_ surface without affecting the tin dioxide (Table 4). It was shown that the correct determination of the element concentrations could be carried out using standard aqueous solutions. It was found that the surface antimony concentration was (1.6 ± 0.3) 10^−2^ wt.%, while the total antimony content was (2.8 ± 0.3) 10^−2^ wt.%. The application of the proposed analysis technique made it possible to determine the composition of semiconductor materials with a standard deviation of s_r_ = 0.10. Using this approach to analyze the composition of nanocrystalline SnO_2_ with Au and Sb additives, it was possible to establish that gold was immobilized on the SnO_2_ surface, while antimony was distributed between the surface and the volume of the SnO_2_ grains [202].

A similar approach using the partial and complete dissolution of nanocomposites and the subsequent analysis of the obtained solutions by the ICP MS method was employed to determine the concentration of chromium with s_r_ = 0.07 and to study its distribution between the volume and the surface of SnO_2_ nanocrystals [90]. In an octahedral oxygen environment, the ionic radius of Cr^3+^ is smaller than that of Sn^4+^ (0.615 Å and 0.69 Å, respectively), which allows the chromium ion to be embedded in the crystal lattice of tin dioxide [203]. To determine the chromium concentration on the surface of SnO_2_ nanocrystals, the samples were treated with potassium rhodanide, after which the obtained solutions containing stable chromium rhodanide complexes were analyzed. The novelty of this approach should be noted, since chromium oxides are barely soluble and are effectively dissolved by fusing them with sodium carbonate and subsequent leaching with an alkali [204]. The chromium content in the crystal structure of SnO_2_ was determined from a solution obtained after the decomposition of the sample residue in a mixture of HCl + HF + HNO_3_ acids in a microwave autoclave. It was shown that, regardless of the amount of modifier introduced during the synthesis, in samples synthesized by the impregnation of the SnO_2_ matrix, 20–30% of the total amount of introduced chromium was on the surface of the materials, and 70–80% was distributed in the crystal structure of the SnO_2_. Using this approach, it was also shown that there was no chromium on the surface of materials synthesized by flame spray pyrolysis, which provides a high degree of mixing of precursors under synthesis conditions. Thus, this method of producing modified tin dioxide ensures the distribution of the additive only in the SnO_2_ crystal structure. The total amount of chromium in the samples was no more than 0.01 wt.%, which was an insignificant portion (1–3%) of the chromium introduced during synthesis. By this fact, the authors explained the deterioration of the sensor properties of materials synthesized by flame spray pyrolysis compared with materials obtained via impregnation.

A study of the distribution of elements in SnO_2_/CoO_x_ nanocomposites carried out by the HAADF-STEM method with EDX mapping showed that in nanocomposites obtained at a low annealing temperature (300 °C) for the SnO_2_ matrix, cobalt was evenly distributed over the surface of the SnO_2_ crystallites, without forming particles of individual cobalt-containing oxide phases [63]. On the contrary, in nanocomposites synthesized using a high annealing temperature (750 °C) for the SnO_2_ matrix, there were both particles containing only tin and particles containing only cobalt. The quantitative analysis of the composition of nanocomposites was carried out by the ICP MS method. To assess the distribution of cobalt between the volume and the surface of SnO_2_ crystalline grains, sample preparation techniques have been developed that allow one to independently determine the cobalt content on the surface of SnO_2_ (without destroying the tin dioxide matrix) and the total cobalt content in nanocomposites (under conditions of the complete dissolution of the SnO_2_ matrix). In nanocomposites annealed at 300 °C, the amount of cobalt found on the surface of SnO_2_ grains does not exceed 30% of its total content in nanocomposites. This allows us to assume that in these samples, a portion of the cobalt is embedded in the SnO_2_ crystal structure. In these nanocomposites, cobalt plays the role of electroactive additive. For samples annealed at 750 °C, the amount of cobalt detected on the surface coincides with the total cobalt content within the error of the analysis, i.e., under these synthesis conditions, cobalt does not enter the tin dioxide crystal structure and is localized as cobalt-containing oxide phases on the surface of SnO_2_ crystalline grains. The formation of *p*-CoO_x_/*n*-SnO_2_ heterojunctions is responsible for the electrical properties of these nanocomposites and determines their sensor properties when detecting CO, NH_3_, and H_2_S.

To analyze the distribution of components in SnO_2_/CoO_x_/Au nanocomposites, in which Au is located on the SnO_2_ surface and cobalt can be distributed between the surface and the volume of the SnO_2_ crystallites [98], a new procedure for determining the concentration of additives separately in the volume and on the surface of SnO_2_ by the ICP MS method has been developed. The essence of the method consists in the preliminary dissolution of gold and cobalt oxide on the surface with a mixture of acids (HNO_3_:HCl = 1:3), centrifugation, and the decantation of the filler solution. The use of this method practically eliminates the transition of tin (which is the main component) into the solution. After dissolving the additives localized on the SnO_2_ surface, the samples are decomposed in a mixture of HNO_3_, HCl, and HF in a microwave oven, followed by the determination of the concentration of Co, Au, and Sn by the ICP MS method [205]. It should be noted that the determination of gold in the content range of 0.03–0.1 wt.% is possible only with the use of the highly sensitive ICP MS method.

A distinctive feature of the considered approaches is the need to transfer difficult-to-dissolve cassiterite into a solution suitable for ICP MS analysis. The successful resolution of this task using SnO_2_ cementation with zinc powder in hydrochloric acid was proposed in [201]. However, such a sample preparation technique is time-consuming and leads to the zinc contamination of the analyzed solutions. The approach of sample decomposition in an autoclave with microwave intensification seems to be more promising. The decomposition of samples in autoclaves within a microwave oven has a number of advantages: the ability to control the temperature and heating modes and no need to use large volumes of reagents, which led to a decrease in the signal of the blank experiment [138,206]. This method was used in [63,64,90,201] for the dissolution of various SnO_2_-based samples. At the same time, the acid mixtures selected for dissolution depend on the type of additives, and the furnace heating programs differ for SnO_2_ samples synthesized at different temperatures. With an increase in the synthesis temperature, the concentration of defects in the nanocrystals decreases, and particles become larger. Therefore, for samples synthesized at temperatures above 600 °C, a specific autoclave heating program is necessary. For example, the complete decomposition of SnO_2_/MnO_x_ nanocomposites requires heating up to 220 °C in two stages for three hours [64]. In other cases, processing in a mixture of HCl + HF (+HNO_3_ for platinum group metals) for 30 min at 180 °C is sufficient to dissolve cassiterite [128].

The results of the separate determination of platinum and palladium concentrations using the previously proposed ICP MS approach were used to compare the synthesis methods of modified nanocrystalline SnO_2_ [207]. The synthesis of the SnO_2_ matrix was carried out by two methods: chemical precipitation and flame spray pyrolysis. Noble metals were introduced by the impregnation of the SnO_2_ matrix with subsequent thermal decomposition. It was established that palladium was located on the surface of the nanocomposites regardless of the synthesis procedure. At the same time, no loss of the additive was noted. It was shown that for SnO_2_ obtained by chemical precipitation, 30–50% of the Pt was distributed on the surface, and the remaining 50–70% was in the volume of the SnO_2_ grains. The use of flame spray pyrolysis for SnO_2_ synthesis increased the Pt content on the SnO_2_ surface by up to 80%. Platinum losses were noted for both synthesis methods, which may have been due to various factors. Based on the low reproducibility of the results of the Pt and Pd determination (s_r_ = 0.35), the heterogeneity of the additives’ distribution in the studied materials was established.

Note that despite the developed procedure for dissolving SnO_2_ for ICP MS analysis, this stage of sample preparation increases the time and complexity of the analysis and can serve as a source of errors. An alternative may be the use of total reflection X-ray fluorescence (TXRF). An important feature of the TXRF method is the formation of standing waves on the surface under conditions of the complete external reflection of X-rays, which contributes to the effective excitation of the X-ray fluorescence of the sample under study. Another feature concerns the registration of characteristic radiation. In TXRF, the detector is placed very close to the surface of the sample holder, providing a large solid angle of radiation collection; effective registration; and, as a result, a high counting speed that reduces the analysis time to 100–1000 s. Due to the use of a thin layer of the sample, there is no phenomenon of X-ray reabsorption. Thus, when the thickness of the sample layer decreases below a certain critical value, matrix effects practically do not appear. In analytical practice, TXRF makes it possible to eliminate the typical disadvantages of energy-dispersive X-ray fluorescence analysis, such as low sensitivity, the presence of matrix effects, and the need for external calibration.

TXRF uses a unified geometry of 0.1°/90° (usually referred to as 0°/90°), and since samples are used for analysis in the form of thin layers, X-ray radiation is weakly scattered. This makes it possible to significantly reduce the level of background radiation and significantly improve the detection limits, reaching in some cases levels of μg/kg (or μg/L). In TXRF, the calculation is usually implemented according to the method of the internal standard, which eliminates the need for the preparation of standard reference samples. This provides great advantages in cases where the preparation of standard samples is difficult, for example, new nanocrystalline materials. In this way, ruthenium in SnO_2_ was determined at a concentration level below 1 wt.% using gallium as an internal standard. At the same time, it was shown that the reproducibility of the results of Ru determination by TXRF was higher when using solutions obtained via sample decomposition than in suspensions without sample preparation (s_r_ = 0.09 and 0.15, respectively), because of the complex morphology of the surface formed after the suspension dried on the reflector substrate. In combination with the low matrix influence, this makes the TXRF method suitable for the analysis of complex objects [208,209,210]. Thus, the authors of [211] developed a method for determining Ru in SnO_2_ suspensions by the TXRF method without sample preparation, while the correctness of the determination results was confirmed by the ICP MS method after the analysis of sample solutions following decomposition. The authors of [211] also noted the limitations of the TXRF method. The definition of Pd was impossible, as the analytical line for Pd L_α_ (2.84 keV) partially intersected the line for Ar K_α_ (2.96 keV) from the air layer between the sample and the X-ray radiation detector. For this reason, the distribution of ruthenium and palladium between the surface and the volume of SnO_2_ nanocrystals was studied by the ICP MS method in solutions obtained after processing samples with concentrated HCl. It was shown that tin did not pass into solution together with Ru and Pd under the selected conditions. It was found that the concentration of Ru and Pd on the SnO_2_ surface was 0.4 and 0.2 wt.%, respectively. It was noted that it was optimal to use the ICP MS method to determine the noble metal additives at a level of less than 1 wt.% in samples of low weight.

According to [180,181], gold clusters are distributed on the surface of SnO_2_; therefore, the use of the TXRF method for sample suspensions significantly simplifies the analysis of the Au concentration in SnO_2_. In this case, it is possible to directly determine the total Au content at the level of 1–2 wt.% using gallium as an internal standard with s_r_ = 0.12 [212]. Interestingly, the results of the analysis of suspensions by the TXRF method, in contrast to ICP MS, can be used to assess the uniformity of the additive distribution over the surface by evaluating the results of determination in individual aliquots taken from the volume of suspensions [213]. For the correct determination of Au at a concentration level of 0.2 wt.%, the sensitivity of the TXRF method is not sufficient due to the need for low weight sampling to meet the conditions of a thin layer when analyzing the sample. Therefore, it is proposed to use another approach to determine such low concentrations.

For SnO_2_-based nanocrystalline materials, a procedure has been developed for the joint and direct determination of Au and Co additives and the Sn macrocomponent [98]. The approach includes the combination of high-resolution continuum source graphite furnace atomic absorption spectrometry (HR CS GFAAS) and the application of sodium carboxylmethylcellulose (Na-CMC) for the preparation of the sample suspension. The direct determination of the analytes is possible in a suspension of powder samples and an aqueous standard solution with s_r_ ≤ 0.04. The proposed technique may be useful for routine laboratory analyses of nanocrystalline materials. The accuracy of the results was proven by ICP MS after sample decomposition and solution analysis [98].

SnO_2_/MnO_x_ nanocomposites prepared by SnO_2_ impregnation with Mn(acac)_3_ and subsequent thermal annealing [64] with various manganese contents (up to [Mn]/[Sn] = 10 mol.%) and different manganese distributions were characterized by TXRF and ICP MS. In this case, as a reagent for removing manganese from the SnO_2_ surface, oxalic acid was used, which formed stable complex compounds with the manganese. The obtained solutions were analyzed by the ICP MS and TXRF methods. The convergence of the results for manganese determination by these methods within an error of 5% was shown. At the same time, it was demonstrated that due to the low manganese content in the volume of the SnO_2_ nanocrystals, the determination of the additive concentration in samples after etching the surface was possible only using the ICP MS method in solutions obtained after sample decomposition in a microwave autoclave. A different distribution of manganese between the volume and the surface of the SnO_2_ crystallites was revealed depending on the total Mn concentration. The Mn (surface)/Mn (total) ratio was 0.6 ± 0.15 and 0.94 ± 0.25 for samples with a composition [Mn]/[Sn] = 1 and 10 mol.%, respectively. Thus, although the manganese was introduced as a surface modifier via the impregnation route, there was a doping effect caused by the redistribution of manganese between MnO_x_ surface segregation and the SnO_2_ crystal structure. The manganese cations introduced in the SnO_2_ lattice acted as electroactive additives. Surface MnO_x_ segregation had a significant influence on the sensor properties of the SnO_2_/MnO_x_ nanocomposites. The presence of manganese in the total concentration of [Mn]/[Sn] = 1 mol.% increased SnO_2_ sensitivity towards CO and prevented signal inversion when detecting NO.

## 6. Conclusions

A detailed and thorough characterization of new nanocrystalline oxide materials used as a sensitive layer in semiconductor gas sensors is a necessary basis for reliably establishing the relationship between synthesis conditions, composition, structure, and sensory parameters (sensitivity and selectivity) in detecting gases of various natures. Whereas the term “composition” is complex and includes the gross quantitative elemental composition, phase composition, surface composition, electronic state of additives and main components, and distribution of additives between the volume and the surface of the metal oxide crystallites, to determine each of the abovementioned constituents, it is necessary to choose an adequate method of analysis. Besides this, the methods for determining the composition of nanocrystalline materials have a number of features. Given the small particle size and complex distribution of the additive in thin near-surface layers, the characterization of nanocomposites should be carried out using complementary methods. Along with X-ray diffraction, important information about the structure and phase composition of such systems can be obtained by scanning and transmission electron microscopy, electron diffraction, Raman spectroscopy, and Mössbauer spectroscopy. Traditional methods of surface investigation (Auger electron spectroscopy and X–ray photoelectron spectroscopy) also provide information averaged over a layer of 2–2.5 nm, which is comparable to the minimum diameter of crystal grains of 3 nm. These methods require the use of high vacuum, which creates additional difficulties in result interpretation. The approach based on the selection of a reagent for transferring an additive immobilized on the surface of tin dioxide to a solution without dissolving the SnO_2_ matrix, followed by the analysis of the obtained solutions by TXRF and ICP MS methods, allows one to determine the additive concentrations on the surface and in the volume of SnO_2_ nanocrystals with high accuracy. It should be noted that, despite the proposed approaches to the decomposition of the SnO_2_ matrix in a mixture of acids using an autoclave, the dissolution conditions, namely the ratio of acid concentrations and the temperature regime, must be adapted to a specific material. This time-consuming stage complicates the analysis procedure. An alternative is the use of multielement TXRF for the analysis of suspensions of undisturbed nanocomposites and the SnO_2_ matrix after flushing additives from the surface. The selection of an internal standard for quantitative calculations makes it possible to reproducibly determine the concentration of the additive at the level of 1 wt.%.

## Figures and Tables

**Table 1:** Comparison of characteristics of analysis methods.

Method	Working Principle	LOD	Analytical Spot Size	Depth of Analysis	Information
Energy-dispersive X-ray spectroscopy (EDX)	Generation of characteristic X-rays from a specimen through the electron beam	0.1–1 at%	≥0.1 μm (combined with SEM) 0.05–0.2 nm (combined with STEM)	0.1–3 μm	Gross elemental composition except for light elements; visualizing element distribution (EDX mapping)
X-ray diffraction (XRD)	Interference of monochromatic X-rays	~1% by volume		~20 Å to ~30 µm depending on material properties and X-ray incidence angle	Phase composition; determining the crystallographic properties of a sample
Raman spectroscopy	Scattering of incident light at an energy shifted by the vibrational energy (hν) of the molecule	100 ppb	~1 µm	<10 µm	Chemical bonding/molecular information; phase composition; surface adsorbates
X-ray photoelectron spectroscopy (XPS)	The simultaneous measurement of kinetic energy and the number of electrons escaping when the sample is irradiated with a beam of X-ray radiation under high vacuum	0.1–1 at%	~10 μm	10 nm	Surface elemental composition; element oxidation state
Inductively coupled plasma mass spectrometry (ICP MS)	Ionization of atoms in plasma and mass selection	1–100 ppt	volume	volume	Determining trace elements (<1%) present in a sample
Total reflection X-ray fluorescence (TXRF)	Energy-dispersive X-ray fluorescence spectrometry on a thin layer of the sample	ng–pg	size of the collimator of the detector (8 mm diameter)	volume	Gross elemental composition except for light elements; determining trace elements (<1%) present in a sample
High-resolution continuum source graphite furnace atomic absorption spectrometry (HR CS GFAAS)	Absorption of ground state-atoms of UV or visible light in the gaseous state	10^−6^–10^−1^%	-	volume	Determining trace elements (<1%) present in a sample

**Table 2:** Methods of introducing additives that provide the possibility of forming the phase of the second component.

Material	Synthesis Method	Ref.
SnO_2_/TiO_2_ nanobelts	I: Hydrothermal (TiO_2_); II: hydrothermal (SnO_2_)	[136]
SnO_2_/TiO_2_ nanotubes	I: Electrochemical anodization (TiO_2_); II: atomic layer deposition (SnO_2_)	[137]
SnO_2_/TiO_2_ nanopowders	I: Sol–gel (TiO_2_); II: sol–gel (SnO_2_)	[138]
TiO_2_/SnO_2_ nanosheets	I: Hydrothermal (SnO_2_); II: pulsed laser deposition (TiO_2_)	[139]
TiO_2_/SnO_2_ nanofibers	Coaxial electrospinning	[140]
TiO_2_/SnO_2_ thin films	I: Magnetron sputtering (SnO_2_); II: Langmuir–Blodgett (TiO_2_)	[141]
V_2_O_5_/SnO_2_ nanowires	I: Vapor–liquid–solid (SnO_2_); II: atomic layer deposition (V_2_O_5_)	[142]
Cr_2_O_3_/SnO_2_ nanofibers	Coaxial electrospinning	[143]
Cr_2_O_3_/SnO_2_ hollow spheres	I: Ultrasonic spray pyrolysis (SnO_2_); II: E-beam evaporation (Cr_2_O_3_)	[144]
Cr_2_O_3_/SnO_2_ nanocomposites	I: Sol–gel (SnO_2_); II: electrodeposition (Cr_2_O_3_)	[145]
Cr_2_O_3_/SnO_2_ nanofibers	I: Electrospinning (SnO_2_); II: impregnation (Cr_2_O_3_)	[146]
MnO_2_/SnO_2_ nanocomposites	Chemical precipitation with subsequent thermal annealing	[147]
SnO_2_/Mn_3_O_4_ nanocomposites	I: Plasmo-chemical deposition (Mn_3_O_4_); II: RF sputtering (SnO_2_)	[148]
Mn_3_O_4_/SnO_2_ nanocomposites	I: Citrate sol–gel (SnO_2_); II: impregnation (Mn_3_O_4_)	[149]
Fe_2_O_3_/SnO_2_ core–shell	I: Chemical precipitation (SnO_2_); II: sol–gel (Fe_2_O_3_)	[150]
Fe_2_O_3_/SnO_2_ hollow spheres	I: Hydrothermal (SnO_2_); II: hydrothermal (Fe_2_O_3_)	[151]
Fe_2_O_3_/SnO_2_ nanocomposites	Citrate sol–gel	[152]
Fe_2_O_3_/SnO_2_ nanorods	I: Hydrothermal (SnO_2_); II: ionic layer adsorption reaction (Fe_2_O_3_)	[153]
Fe_2_O_3_/SnO_2_ nanofibers	I: Electrospinning (SnO_2_); II: hard-template method (Fe_2_O_3_)	[154]
Fe_2_O_3_/SnO_2_ nanofibers	Electrospinning	[155]
Co_3_O_4_/SnO_2_ nanocomposites	I: Chemical precipitation (SnO_2_); II: impregnation (Co_3_O_4_)	[63]
Co_3_O_4_/SnO_2_ nanowires	I: Vapor–liquid–solid (SnO_2_); II: DC sputtering (Co_3_O_4_)	[156]
Co_3_O_4_/SnO_2_ nanowires	I: Vapor–liquid–solid (SnO_2_); II: sol–gel (Co_3_O_4_)	[157]
NiO/SnO_2_ nanowires	I: Vapor–liquid–solid (SnO_2_); II: atomic layer deposition (NiO)	[158]
NiO/SnO_2_ nanosheets	I: Hydrothermal (SnO_2_); II: hydrothermal (NiO)	[159]
NiO/SnO_2_ nanocomposites	I: Chemical precipitation (SnO_2_); II: sol–gel (NiO)	[160]
NiO/SnO_2_ hollow spheres	I: Hydrothermal (SnO_2_); II: pulsed laser deposition (NiO)	[161]
CuO/SnO_2_ hollow nanofibers	Electrospinning	[162]
CuO/SnO_2_ thin films	I: RF magnetron sputtering (SnO_2_); II: RF magnetron sputtering (CuO)	[163]
ZnO/SnO_2_ rootstock/scion	I: Vapor–liquid–solid (SnO_2_ or ZnO); II: vapor–liquid–solid (ZnO or SnO_2_)	[164]
ZnO/SnO_2_ nanofibers	Electrospinning	[165]
ZnO/SnO_2_ nanoheterostructures	I: Hydrothermal (SnO_2_); II: chemical bath deposition (ZnO)	[166]
SnO_2_/Nb_2_O_5_ core–shell	I: Hydrothermal (Nb_2_O_5_); II: atomic layer deposition (SnO_2_)	[167]
Nb_2_O_5_/SnO_2_ nanocomposites	I: Spin coating (SnO_2_ nanosheets); II: hydrothermal (Nb_2_O_5_ nanorods)	[168]
Nb_2_O_5_/SnO_2_ nanocomposites	I: Chemical precipitation (SnO_2_); II: impregnation (Nb_2_O_5_)	[115]
RuO_2_/SnO_2_ nanopowders	I: Chemical precipitation (SnO_2_); II: impregnation (RuO_2_)	[169]
RuO_2_/SnO_2_ nanopowders	I: Chemical precipitation (SnO_2_); II: deposition–precipitation (RuO_2_)	[120]

**Table 3:** Methods of the synthesis of multicomponent nanocrystalline materials that benefit solid solution formation.

Material	Synthesis Method	Ref.
SnO_2_(Sb) thin films	Sol–gel spin coating	[83]
SnO_2_(Sb) nanopowders	Hydrothermal	[84]
SnO_2_(Sb) nanowires	Sb-ion implantation	[85]
(SnO_2_:TiO_2_) thin films	Pulsed laser deposition	[87]
(SnO_2_:TiO_2_) nanopowders	Co-precipitation	[88]
(SnO_2_:TiO_2_) nanopowders	Flame spray pyrolysis	[89]
(Cr_2_O_3_/SnO_2_) nanopowders	Flame spray pyrolysis	[65]
SnO_2_(Mn) nanobelts	Thermal evaporation	[91]
SnO_2_(Mn) thin films	Spray pyrolysis	[92]
SnO_2_(Mn) nanopowders	Co-precipitation	[93]
SnO_2_(Fe) thin films	Spray pyrolysis	[96]
SnO_2_(Fe) nanopowders	Co-precipitation, hydrothermal	[95]
SnO_2_(Fe) nanopowders	Co-precipitation, hydrothermal	[97]
SnO_2_(Co) thin films	Spray pyrolysis	[96]
SnO_2_(Co) thin films	Spray pyrolysis	[100]
SnO_2_(Co) nanopowders	Co-precipitation, hydrothermal	[99]
SnO_2_(Co) inverse opal	Ultrasonic nebulizing deposition with a self-assembly template	[101]
SnO_2_(Ni) thin films	Spray pyrolysis	[96]
SnO_2_(Ni) nanopowders	Co-precipitation, hydrothermal	[95]
SnO_2_(Ni) porous structures	Co-precipitation, hydrothermal	[103]
SnO_2_(Ni) nanorods	Co-precipitation, hydrothermal	[104]
SnO_2_(Ni) nanopowders	Co-precipitation, microwave treatment	[105]
SnO_2_(Cu) porous structures	Surfactant-assisted co-precipitation	[107]
SnO_2_(Cu)/rGO nanocomposites	Solvothermal	[108]
SnO_2_(Cu) nanopowders	Co-precipitation	[109]
SnO_2_(Zn) thin films	Spray pyrolysis	[112]
SnO_2_(Zn) nanostructures	Co-precipitation, hydrothermal	[110]
SnO_2_(Nb) nanopowders	Co-precipitation, hydrothermal	[114]
SnO_2_(Nb) nanopowders	Flame spray pyrolysis	[113]
SnO_2_(Ru) nanopowders	Co-precipitation	[120]
SnO_2_(Ru) nanofibers	Electrospinning	[117]
SnO_2_(Ru) nanotubes	Electrospinning	[118]
SnO_2_(Ru) thin films	Spray pyrolysis	[119]

**Table 4:** Examples of reagents and treatment conditions for flushing the additive from the SnO_2_ surface without affecting the tin dioxide matrix.

Additive on SnO_2_ Surface	Reagent, Treatment Conditions	Analysis Method
Sb^3+^	10% citric acid, heating for 30 min in an ultrasonic bath.	ICP MS, TXRF
Cr^3+^	1% KSCN in 25% NH_3_ with the formation of NH_4_[Cr(NCS)_4_(NH_3_)_2_].	ICP MS, TXRF
Co^2+^, Co^3+^	HNO_3_:HCl = 1:3.	ICP MS, TXRF
PtO_x_, PdO_x_, Au^0^	HNO_3_:HCl = 1:3.	ICP MS
RuO_x_	Oxalic acid or ascorbic acid (pH = 3), HNO_3_:HCl = 1:3.	ICP MS
MnO_x_	Oxalic acid (pH = 3).	ICP MS, TXRF

## Data Availability

The data that support the findings of this study are available from the corresponding author upon reasonable request.
